# Plasma IL-33 levels and immune activation in HIV-TB coinfection: a cross-sectional study in Yaoundé, Cameroon

**DOI:** 10.11604/pamj.2023.46.13.41152

**Published:** 2023-09-11

**Authors:** René Ghislain Essomba, Rostand Munkam Mbe, Marie Paule Ngogang, Claire Bitchong Ekono, Valentina Josiane Ngo Bitoungui, Nassif Seni, Philippe Salomon Nguwoh, Pulchérie Thérèse Ateba, Severin Donald Kamdem, Justin Komguep Nono, Myriam Sylvie Ambomo, Marie Claire Okomo Assoumou, François Xavier Mbopi-Kéou

**Affiliations:** 1 National Public Health Laboratory (NPHL), Ministry of Public Health, Yaoundé, Cameroon; 2 Faculty of Medicine and Biomedical Sciences (FMBS), University of Yaoundé I, Yaoundé, Cameroon; 3 School of Health Sciences, Catholic University of Central Africa, Yaoundé, Cameroon; 4 Faculty of Medicine and Pharmaceutical Sciences (FMPS), University of Douala, Douala, Cameroon; 5 Pneumology Service, Jamot Hospital of Yaoundé, Yaoundé, Cameroon; 6 Faculty of Medicine and Pharmaceutical Sciences (FMPS), University of Dschang, Dschang, Cameroon; 7 Institute of Medical Research and Medicinal Plant Studies (IMPM), Ministry of Scientific Research and Innovation, Yaoundé, Cameroon; 8 Division of Immunology, Health Science Faculty, University of Cape Town, Cape Town, South Africa; 9 Division of Microbiology and Immunology, Department of Pathology, University of Utah School of Medicine, Salt Lake City, Utah, United States of America

**Keywords:** Interleukin-33, HIV, tuberculosis, coinfection, Cameroon, Africa

## Abstract

**Introduction:**

HIV-1 and Mtb are characterized by immune activation and unbalances production of cytokines, but the expression of IL33 in HIV/TB coinfection remain understudied. This study aimed to evaluate the level of IL-33 in plasma of HIV and M. tuberculosis (HIV/TB) coinfected patients compared to patients with respective mono infections in Yaoundé.

**Methods:**

a cross-sectional study was conducted among patients attending the pneumology service and HIV treatment center of the Yaoundé Jamot Hospital. Plasma samples of 157 HIV/TB coinfected patients (n =26, 50% males and 50% females, mean age 39), HIV-1 monoinfected patients (n = 41, 41% males and 59% females, mean age 35), TB monoinfected patients (n = 48, 56% males and 44% females, mean age 37) and healthy controls (n = 42, 29% males and 71% females, mean age 32) were examined by enzyme-linked immunoassay (ELISA) to detect the levels of IL-33 cytokine.

**Results:**

plasma level of IL-33 were higher in HIV/TB coinfected (33.1±30.9 pg/ml) and TB monoinfected individuals (15.1±2.9 pg/ml) compared to healthy controls (14.0±3.4 pg/ml) and could not be detected in most of the HIV-1 monoinfected individuals (12.6±8.7 pg/ml). Interestingly, the increased plasma level of IL-33 in HIV/TB coinfected patients showed a statistically significant difference between healthy controls (33.1±30.9 pg/ml vs 14.0±3.4 pg/ml, P<0.0001) and HIV-1 monoinfected patients (33.1±30.9 pg/ml vs 12.6±8.7 pg/ml, P=0.0002). We further found that IL-33 was higher in patients with high viral load group (40.6±59.7 pg/ml vs 12.6±1.8 pg/ml), P= 0.47) whereas patients under highly active antiretroviral therapy (HAART) showed decreased level of IL-33 concentration as the number of years under ART increased. Our data showed a positive association between plasma IL-33 and viral load in the context of HIV/TB coinfection in our study population with a positive Pearson coefficient of r=0.21.

**Conclusion:**

this study indicates that plasma level of IL-33 differs among HIV/TB coinfected patients and respective monoinfections patients. The increased level of plasma IL-33 reveals that IL-33 measurement in HIV-1 monoinfected patients may represent an early predictor of development of tuberculosis.

## INTRODUCTION

Tuberculosis is a chronic infectious disease caused by *Mycobacterium tuberculosis* (*Mtb*), which is transmitted via aerosols from close contacts with tuberculosis patients. HIV infection has emerged as the principal cause of human immunodepression exposing people living with HIV to opportunistic diseases such as *Mtb*. The geographical overlapping of the two diseases had increased the number of cases of HIV/TB co-infection causing an immense and continuous burden on public health systems, mostly in Low and Middle Income Countries (LMIC) [1]. In fact, people living with HIV are about 30 times more likely to develop active TB, due to compromised immunity [2]. The Joint United Nations Programme on HIV/AIDS (UNAIDS) has reported in 2016 that one million people died of opportunistic infections out of the 36,7 million people living with HIV [3]. Cameroon is classified among the most affected countries by HIV/AIDS in Central Africa. The prevalence rate is estimated at 4.3% and women and children are the most vulnerable [4]. In Cameroon, it is approximatively 39% of all the tuberculosis cases that were positive to an infection with HIV in 2014 [5]. Currently, it is well known that both HIV and tuberculosis (TB) have a profound effect on the immune system and are characterized by a dysregulation of the normal balance of cytokines and the functioning of the cytokine network. The imbalance of cytokine secretion in HIV infection affects the function of the immune system and the course of the disease, increasing or suppressing viral replication [6]. Numerous data indicate that, despite effective antiretroviral therapy (ART), there is evidence of persistent viral production and immune activation in HIV-infected individuals [7-9]. IL-33 exerts multiple effects on immune cells, and is considered as a key player in the control of viral, bacterial and even parasitic infections. IL-33 is a member of the Il-1 superfamily of cytokines and perform its role as an alarm signal in response to cellular damage induced by infection or injury to alert immune cells expressing the receptor [10]. The nature of the antiviral immune response orchestrated by IL-33 depends on the site of infection, the duration and the cytokine environment [11]. A series of studies followed various parameters in HIV/TB coinfection as compared to HIV monoinfection to conclude that *Mtb* and HIV act in synergy, accelerating the decline of immunological functions and leading to subsequent death if untreated [12-14]. Therefore, the analysis of cytokines expressed during a HIV/TB coinfection may be important for predicting the course of the disease. While some studies have already been focused on the evaluation of the expression profile of IL-33 in hepatic disease during schistosomiasis [15], hepatitis, malaria [16], HIV infected patients [17] and others on tuberculosis [18], very few studies have been carried out on the expression of IL-33 in HIV/TB coinfection compared to each of the mono infections [19-20]. The goal of this work was to study the expression of IL-33 cytokine in the context of HIV/TB coinfection and to compare to patients with HIV-1 and TB monoinfections.

## METHODS

**Study design and settings:** the study was a cross-sectional study that was carried out at the Jamot Hospital of Yaoundé, located in the Yaoundé I subdivision, Mfoundi Division, in the Center Region of Cameroon. This health facility is a reference center for treatment and care for both HIV and TB-infected patients in-country [21].

**Study population:** the study population was mainly composed of patients from different population pools at the Jamot Hospital of Yaoundé who voluntarily agreed to participate in the study. These included HIV/TB coinfected patients (n = 26) and TB monoinfected patients (n = 48) who were recruited in the pneumology service of the hospital, HIV-1 monoinfected patients (n = 41) recruited at the HIV treatment service of the hospital and healthy controls (n= 42) from the general population were recruited for the study at the blood bank of the same hospital. Healthy controls repeatedly tested negative for HIV-1 and had no history of TB or exposure to the disease within the past 6 months.

**Data collection:** informed sessions were done in the presence of patients to clearly explain the objective and the methodology of our study. After obtaining the informed consent, a standardized questionnaire was administered to collect demographic and clinical data. Patients with HIV/TB coinfection were naïve for ART and anti-tuberculosis therapy, HIV-positive patients were under first-line ART regimen (tenofovir disoproxil fumarate (TDF) + lamivudine (3TC) (or emtricitabine, FTC) + efavirenz (EFV) 600 mg), TB- positive patients were under first-line drugs (isoniazid, rifampicin). TB diagnoses were based on clinical symptoms, sputum microscopy and TB-Lamp (Tuberculosis-Loop-Mediated Isothermal Amplification) method. The patients were diagnosed as HIV-positive using Rapid Diagnostic Tests (RDT) following the in-country HIV national algorithm.

**Laboratory analysis:** sputum samples were stained for acid-fast bacilli and were graded by light microscopy. Cultures were examined weekly until positive for visible colonies or for a maximum of 8 weeks. Plasma was isolated according to the standard procedure. The whole blood was collected in a vacutainer with EDTA and centrifuged at 1000 rpm for 15-20 min with cooling. The plasma was collected, aliquoted, and stored at -80°C at the National Public Health Laboratory until further analysis. The plasma level of IL-33 was measured using ELISA Kit from BioLegend, Inc. USA (catalog number 435907). The test was performed on undiluted plasma following the manufacturer recommendations. The cytokine concentration was determined using a standard curve obtained with the standards provided by the manufacturer (sensitivity 4.14 pg/mL), and the results were expressed in pg/mL.

**Statistical analysis:** the nonparametric T-test was used to compare two independent groups. The Kruskal-Wallis test was used to compare more than two independent groups. The correlation was evaluated using Spearman´s rank correlation test. The data were analyzed using GraphPad Prism v9.5.0 (GraphPad Software, Boston, MA, USA). Values of p < 0.05 were considered statistically significant.

**Ethical consideration:** all individuals were over 18 years old and gave written informed consent for participation in the study. According to the General Data Protection Regulation (GDPR) requirements, all participants were deidentified and anonymized by assigning them unique codes expressed as an identifier. All clinical samples, data, and study results were stored in an anonymized form. The study was conducted according to the guidelines of the Declaration of Helsinki and approved by the Institutional Ethics Committee for the Research in Human Health of the School of Health Sciences (approval No.2020/01028/CEIRSH/ESS/MIM).

## RESULTS

### Characteristics of the study population

**Figure 1:**
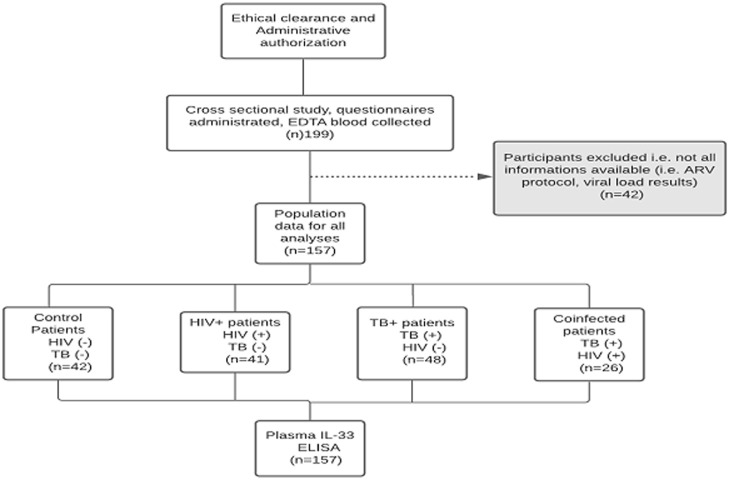
flow diagram describing the strategy of enrolment and examination of 157 patients from Yaoundé Jamot Hospital (YJH), Cameroon

The participants and samples used for the present study were defined as indicated (Figure 1). Samples of participants missing any test result were excluded i.e., missing samples of blood or sputum, ART regimen, viral load results. A total of 157 patients were registered and divided into four different clusters of healthy control, HIV-1 monoinfected, TB monoinfected and HIV/TB coinfected patients. Out of 157 participants enrolled in the study, 26 patients were HIV/TB coinfected patients, 41 HIV-1 monoinfected patients, 48 TB monoinfected patients and 42 healthy controls. The participants' distribution by age, gender and viral load for HIV-positive infected participants are presented below (Table 1). The groups did not differ in demographic indicators. Females predominated (56%) compared to the male gender (43.9%) and the HIV/TB coinfected group had the highest mean age 38.7± 8.6 years. The median viral load in HIV/TB coinfected patients was high (4.22 log10 copies/mL) as compared to that in patients with HIV alone (2.9 log10 copies/mL).

**Table 1:** characteristics of the study population

	Study groups N (%)			
	CONTROL 42(26.7)	HIV (+) 41 (26.1)	TB (+) 48 (30.5)	HIV/TB 26(16.6)
**Age**				
**Mode [Range]**	34[22-52]	31[24-59]	29[23-61]	39[25-57]
**Mean (SD)**	31.7 (7.7)	35.4 (8.2)	36.7 (11.3)	38.7 (8.6)
**Gender**				
**Male/Female**	12/30	17/24	27/21	13/13
**Viral load (log10 copies/mL), IQR**	-	2.9 (1.7-3.4)	-	4.2 (2.6-5.8)

### Plasma levels of IL-33 in patients with HIV/TB coinfection compared to HIV and TB monoinfections

**Figure 2:**
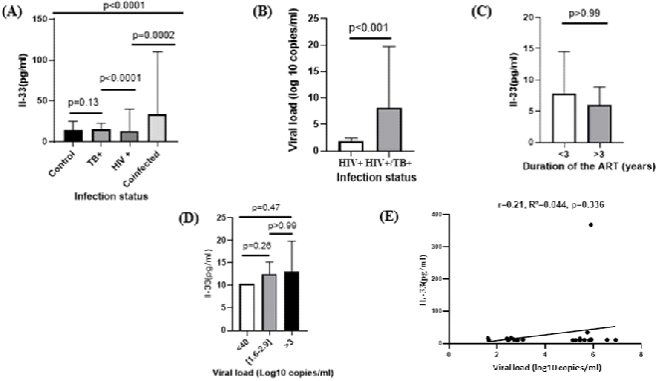
plasma levels of IL-33 in patients with HIV/TB coinfection, HIV monoinfection, TB monoinfection, and healthy controls. A) comparative graph of plasma level of IL-33 in HIV/TB coinfection, HIV monoinfection, TB monoinfection individuals cataloged according to their infectious status. B) HIV viral load of all enrolled participants with HIV and HIV/TB coinfection. C) plasma level of IL-33 according to antiretroviral treatment duration in HIV-monoinfected individuals. D) plasma level of IL-33 according to the viral load in HIV/TB coinfected participants. E) correlation between IL-33 and viral load in HIV/TB coinfected participants

To assess whether plasma levels of IL-33 were different from one group to another, plasma IL-33 concentrations were represented for all participants according to their infection status as reported in Figure 2, A. All four patient´s groups differed in the levels of IL-33 expression, with the major differences observed between the patients with TB monoinfection and HIV/TB coinfection. IL-33 secretion in the group of patients with HIV/TB coinfection who were naïve to anti-tuberculosis and antiretroviral therapy was increased by 2.6 times, and 2.4 times compared to the group of patients with HIV alone (33.1 ± 30.9 pg/ml vs 12.6 ± 8.7 pg/ml, p=0.002) and controls (33.1 ± 30.9 pg/ml vs 14.0 ± 3.4 pg/ml, p<0.0001) respectively (Figure 2, A). Comparison of viral load between HIV/TB coinfected participants versus HIV monoinfected participants presented in (Figure 2, B) showed a very high viral load in HIV/TB co-infected participants compared to the other group (8.24 ± 11.4 log10 copies/mL vs 1.7 ± 0.7 log10 copies/mL, p<0.001). Furthermore, we studied the association of levels of IL-33 with disease progression. We divided patients into three subgroups according to their viral loads: low viral load (<40 log10 copies/mL), medium viral load (1.6-2.9 log10 copies/mL) and high viral load (>3 log10 copies/mL). The results showed that IL-33 was higher in patients with high viral load group (69.1 ± 25.3 pg/ml) as compared to those with medium viral load (12.6 ± 1.8 pg/ml ) and low viral load ( 40.6 ± 59.8 pg/ml) (Figure 2, D). Interestingly, although the differences were not statistically significant, there was a tendency for plasma IL-33 levels to decrease as the number of years under ART therapy increased (3.7 ± 6.7 pg/ml vs 3.6 ± 2.8 pg/ml, p>0.99) (Figure 2, C). This was convincingly substantiated by the observation that plasma IL-33 levels increased with viral load in HIV/TB coinfected individuals clearly establishing the positive association between plasma IL-33 and viral load in the context of HIV/TB coinfection in our study population (r=0.21, p=0.34; Figure 2, E).

## DISCUSSION

A full understanding of the pathogenesis of HIV/TB coinfection has yet to be achieved. In the present study, to gain better understanding of the role of IL-33 cytokine in immunopathogenesis of HIV/TB coinfection, we aimed to determine whether HIV/TB coinfected patients compared to patients with respective mono infections are associated with alterations in IL-33. The results of our study showed that plasma levels of IL-33 were elevated in HIV/TB coinfected and TB monoinfected patients compared to healthy controls and could not be detected in most of the HIV-1 monoinfected individuals.

Both HIV-1 infection, even when successfully treated, and *Mtb* infection are characterized by chronic inflammation. Inflammation is a nonspecific response to pathogens that involves multiple cells, tissues, and organs. While acute inflammation is stopped, chronic inflammation persists, leading to a chronic inflammatory disease characterized by an overproduction of proinflammatory cytokines and recruitment of inflammatory cells (neutrophils and monocytes) to the affected area(s) [20]. Thus, *Mtb* and HIV-1 exploit different scenarios of immune hyperactivation/chronic inflammation. In this study, we have addressed their overlap in drug-naïve patients in the context of HIV/TB coinfection, HIV-1 infected patients under ART and *Mtb* under first-line drugs in a resource-limited setting. To the best of our knowledge, no data have clearly explained the expression of IL-33 plasma levels in the context of HIV/TB infection in Africa.

The first main finding of this study was that HIV/TB coinfected and TB monoinfected patients showed an up-regulated concentrations of IL-33. IL-33 is a cytokine of the IL-1 family with pro-inflammatory and anti-inflammatory effects in response to infection [22]. IL-33 attracted attention because, aside from its traditional role as an “alarmin,” it is known to be involved in innate and adaptive immune responses by enhancing natural killer, Th2, CD4 and CD8 T-cell functions which play a central role in the protective immunity against *Mtb* and HIV infection [19]. In our study, HIV/TB coinfection was characterized by high plasma levels of IL-33, which were increased 2.6-fold and 2.4 fold compared to the levels in patients with HIV-1 and TB monoinfections indicating the additive effects of the two pathogens on IL-33 production. In this way, HIV-induced production of IL-33, protective for HIV-1 patients under highly active antiretroviral therapy, appeared to be detrimental for naïve HIV-1/TB coinfected patients, contributing to HIV-1 persistence and tissue damage in the context of HIV/TB coinfection. Previous studies on the alterations of IL-33 in HIV and TB -infected patients showed inconsistent conclusions [11,17,23-25]. We found that the serum level of Il-33 was slightly higher in the TB monoinfection patients compared to healthy controls, although no statistically significant difference was observed between the two groups, which is consistent with previous research [25]. Perhaps this was due to normal variation above the level of IL-33, which could become significant with increasing the sample size. It should also be considered that the control group included in this study had no history of infection or inflammation, which in turn affects IL-33 levels. It was also found that the expression of IL-33 levels was not increased in HIV infected patients compared to the healthy controls, which is contrary with previous research [24]. The elevation of IL-33 has been observed in a number of types of cancers [26,27], in COVID-19 infection [28] and in chronic hepatitis B infection [29]. Our study was consistent with Miyagaki *et al*. and Mehraj *et al*. studies, which didn't find the elevated expression of IL-33 in HIV infected individuals. However, our finding was not consistent with Wu *et al*. study which found elevated expression of IL-33 in HIV infected individuals. All these studies used ELISA kit to detect the expression levels of IL-33, so the methodology may not be the cause of the different results. We postulated that the differences in clinical characteristics of patients may lead to the different findings in these studies. For example, our study as well as Miyagaki *et al*. and Mehraj *et al*. studies enrolled HIV infected patients under ART, while the HIV infected patients enrolled in Wu *et al*. study were naïve HIV infected individuals. This can be explained by the fact that plasma obtained from HIV-infected individuals under ART display constitutive production of biologically active IL-1α and IL-1β proteins, which are suppressed during the course of antiretroviral therapy [22,23].

We then studied IL-33 expression level with respect to disease progression in HIV infected individuals. Although IL-33 can enhance immune response function, we observed that the increased IL-33 level in naïve HIV/TB coinfected individuals correlates positively with viral load which was consistent with previous research [17]. We postulated that the function of IL-33 in naïve HIV/TB coinfection may be masked due to the complex immune regulation. Additionally, HIV-1 patients under highly active antiretroviral therapy (HAART) showed decreased level of IL-33 concentration as the number of years under ART increased. This is in accordance with the data of other researchers who received similar results [24]. A previous study has shown that mucosal barrier tissues such as gastrointestinal tract store large amounts of IL-33 that may be released upon tissue injury [30]. In HIV infection, gut damage occurs early after infection [31], which can cause the elevation of IL-33 and sST2. Most likely that gut tissue damage in early HIV infection are linked to the elevation of IL-33 observed in naïve HIV/TB coinfected individuals in our study.

This study has a major strength that must be highlighted. Since 2013, the public health policies in Cameroon recommend ART administration as soon as possible after the diagnosis of HIV positivity. Therefore, the HIV+ individuals evaluated in this study present clinical conditions related to infection and clinical progression which are now rare and very difficult to obtain in new studies involving HIV+ populations. As potential limitations of this study, we have to consider (i) the sample size, (ii) we did not exclude parasitic diseases that can also increase or reduce the production of our chosen cytokine, (iii) we did not have the opportunity for a long term follow-up of patients, (iv) the evaluation of a single cytokine instead of a combination of several cytokines profiles. This type of data is useful to indicate general patterns of immune activity and Th1/Th2/Th17 balances. Future studies will be conducted on a wider group of patients and will include a detailed characterization of the Th1, Th2 and Th17 cytokines in plasma of patients to attribute the observed profiles of cytokine expression as well as to correlate it with the clinical course of both infections.

## CONCLUSION

We have shown that naïve HIV/TB coinfected patients are characterized by increased plasma levels of IL-33, which is crucial for the control of both infections. The increased levels of plasma IL-33 indicate that IL-33 measurement in HIV-1 monoinfected patients can be a potential surrogate marker of immune activation in the context of HIV-TB coinfection in a resource-limited setting. This data broadens the understanding of HIV pathogenesis and provides critical information for HIV intervention.

### What is known about this topic

- *Both HIV and tuberculosis (TB) have a profound effect on the immune system and are characterized by a dysregulation of the normal balance of cytokines and the functioning of the cytokine network;*
- *Elevation of IL-33 has been observed in a number of types of cancers, in COVID-19 infection, in hepatic disease during schistosomiasis, in malaria and in chronic hepatitis B infection;*
- *Previous studies on the alterations of IL-33 in HIV and TB -infected patients showed inconsistent conclusions*.

### What this study adds

- *This study shows the upregulation of IL-33 cytokine in plasma in the context of HIV/TB coinfection compared to patients with respective mono infections;*
- *The increased level of IL-33 in HIV/TB coinfected individuals correlates positively with the viral load;*
- *The measurement of IL-33 in HIV-1 monoinfected patients may represent an early predictor of development of tuberculosis*.
